# The Scotch Introductory Addresses

**Published:** 1881-04

**Authors:** S. D. Campbell Black


### THE SCOTCH INTRODUCTORY ADDRESSES—BY S. D. CAMPBELL BLACK.

If we discard the technical introductory addresses recently delivered in the Scotch Universities by one or two of the professors, such as those of Professor Cleland and Charteris at Glasgow, and Professor Turner at Edinburgh, it is remarkable with what unanimity the other professors assailed—and it is significant of the times —what they are pleased to term, and vexed evidently to recognize as, the materialistic and rationalistic current of popular opinion at present. As a matter of course two of the clearest thinkers and most elegant writers of the day, Huxley and Spencer, came in for a fair share of attention, and the customary vituperation. Now this a matter which falls quite competently within the domain of the physician—particularly of the medical psychologist—especially as the proneness of medical men to scepticism, if not absolute Atheism, is unjustly proverbial, and is accepted as merited in quarters where we should not have anticipated it. The church in Scotland, and those immediately dependent on the perpetuation of ecclesiasticism, are profoundly agitated at present in endeavors to reconcile their means of living with the dawning intelligence of the multitude, and the perplexities of many are rendered still more inextricable by the ingenuity which education commands, and is not slow to employ in this process of reconciliation. It is too well known to practitioners of medicine to what a painful extent mental aberration is due to religious perplexity of some kind or another, and it is much to be doubted whether the well-meaning attempts alluded to are calculated to obviate this contingency. The question presents, Is it not compatible with a happy and useful existence—with even a Godly existence—to cast aside that which in the nature of things lies of necessity beyond the domain of sense and reason, which is, therefore, thus incomprehensible, and of which no amount of empty and high-sounding talk will ever enable humanity, as at present constituted, to take cognizance? Are there not thousands of men and women, too, of of spotless reputation who do this, and avow it; and as many who secretly think thus, but whose avowal thereof is restrained by social considerations, or false and pusillanimous notions of propriety? For too long a time mythology and morality have been regarded as inseparable. It were well if people reflected a little more that the ultimate tribunal to which all our knowledge is referable is that of *human observation and experience*, and all our conceptions of the external world (and we can have no other) are appreciated alone through the medium of our senses. If this undoubted and unassailable position were more frequently kept in view, from what an amount of inconsequent and illogical rubbish would society be exempt. Professor Blackie, of Edinburgh, is a versatile man, a good linguist, an indifferent rhymer, and, in his own opinion, at least, an authority on most subjects. In an address, on the occasion of the the opening of his class, "On Science and Literature in their Relation to Culture," he indulged in the now customary fling at Huxley and physical science, with what success a quotation or two from his address will show: "Whatever discoveries may have been made with regard to the composition of Professor Huxley's favorite primordial substance called protoplasm, or the special functions of this or that convolution of the brain, or the missing link between any type of curious buckler-headed fishes in the Devonian bed, and the rich crop of tadpoles and toads in the Triassic strata, it will remain an impossibility that any science which concerns itself chiefly with the shell or pulp of inferior organisms divorced from vitality can be so interesting to human beings as the soul of living man. For it lies in the very nature of physical science that it occupies itself principally with things from which life is gone, or, at least, which cannot be examined when the living processes are in a complete state of normal exercise." . . . "God is life and God is love. Of this prime source of all human greatness mere physical science can teach nothing." Professor Blackie is inexcusably in error in thinking that physical science takes not equally, if not greater cognizance of the living than of the dead organism. It sees in the living the completion and the perfection of organization; from the dead it deduces conclusions as to the living within the domain of sense and reason, and in harmony, as already stated, with human observation and experience. Further it does not pretend to go, and with as much ardor and reverence as professor Blackie, it regards, in the best sense of the term, the proper study of mankind to be man. That which physical science regards as the sum of the phenomena of healthy cerebration, the "Modern Athenian" maintains to be a distinct entity, whose study is to be specially relegated to what he calls "moral science," "the science directly of the living man;" and this science, according to him, "is known not by peeping microscopes or far-sweeping telescopes." The interdependence of the *mens sana* on the *corpus sanum* duly acknowledged by his ancient friends, is at least inferentially ignored by the learned Professor. Physical science embraces the study of men, animals, and Nature as a whole—their attributes as well as their material. Blackie's "moral science" comprehends but the cerebral manifestations of man. Professor Blackie affects a fondness for "exact definition;" would the learned gentleman oblige us by an exact definition of his "soul," or "mind," which we hope we are not wrong in assuming to be synonymous terms? Will he put into understandable English any idea which is not a material reflection, so to speak, *primarily* conveyed to the cerebrum through educated sense? If any one assert that the sun is inhabited by human beings similar to those which inhabit this planet, we naturally smile at the innocence of the statement, and ask the proof thereof, sceptical of its being forthcoming, as being a violation of reason and experience; for with the exception of three ancient Hebrews, into the salamander composition of whose bodies we do not stop to inquire at present, the surrounding medium of fire is not *usually* compatible with the existence of organized vital structure. The *onus probandi*, therefore, lies with him who makes any statement which violates reason and sense. The special beverage of his friend the Celt, as the Professor may be assumed fairly to understand, possesses a singular disturbing influence on cerebral circulation, *pari passu*, it effects the soul, or mental functions; even the Professor has been known to sing, if not to dance, in presence of this "holy ecstacy;" it may be pushed so far as to cause coma, from which recovery may take place, but during this state the mental functions are in complete abeyance—the mind has been a complete blank. Should the molecular changes caused in the brain by the circulation of blood, and of which thought is the expression, be permanently arrested, and its organic structure become a prey to decomposing chemical changes, what has become of the mental phenomena, and what proof have we of their existence apart from vitality? These are questions not to be shirked, or ruthlessly pushed aside by anyone owning a philosophic allegiance to truth.

The colleague of the learned Profcsssor of Greek, Professor Charteris (Edinburgh), from his coign of vantage, leveled his ecclesiastical thunderbolts at "Rationalism as Antagonistic to Christianity." By "rationalism," he understood "the doctrine or the tendency of those who admitted nothing they could not understand or at least clearly reconcile with reason." The definition is not a fair one. The rationalist is on every hand surrounded by mysteries hidden deep in the bosom of Nature, whose existence is to him indisputable, but which unaided sense fails him in unraveling. The tiny seed, he knows, retains its vitality for thousands of years; of this he has proof afforded by the evidence of his senses; and he believes that, from the dawn of time, the gigantic heavenly spheres observe their magnificent course with a regularity and grandeur whose contemplation prostrates his highest aspirations in the dust. These things he cannot understand; he knows they exist; he can formulate no reasonable solution of their genesis nor maintainance, but he is content to know, and humbly to feel, his ignorance, rather than create any theory which would violate his cultivated reason. Professor Charteris indicated the common possession of this "dreadful materialism" and Christianity, ingeniously claiming for the latter a "reverence for facts," and adding that "it would be a bad day for Christianity when it ceased to claim that as its distinction, amid the many forms of faith and unbelief to which men adhered." It is no business of ours to contend with the amiable professor on this subject; but tried by the standard above submitted, and a "reverence for facts," we would be curious to learn in what form Christianity would present after having undergone the ordeal. Religion without a reasonable basis, Professor Charteris seems to admit to be worthless; and there is no avoiding the conclusion, faith without some show of reason is simple superstition.

What concerns us as medical practitioners is the care of many of unstable mental equilibrium rendered unfit for their duty to themselves and to society by vain and hopeless striving to comprehend mysteries in religion unfathomable, and in Nature incomprehensible. Is it not compatible with their well-being, and our duty to society that we enjoin, at least, a suspense of judgment on questions beyond the domain of sense and reason; inculcate the beauty of morality, and the manliness af truth; the seeking of good for its own sake, and the shunning of evil as incompatible with a happy and useful existence; and to impress upon such as are thus influenced that it were an outrage on Deity that he can be the Nemesis depicted, especially from Scotch pulpits.

